# Trends and outcomes of thoracoscopic esophageal atresia and tracheoesophageal fistula repair: a retrospective analysis 2016–2022

**DOI:** 10.1007/s00383-026-06420-8

**Published:** 2026-04-15

**Authors:** John M. Woodward, Patricia Corujo Avila, Aaron Orellana, Kaity Tung, Melanie Tacher Otero, Lindsey Caines, Krystle Bittner, Anshul Kumar, Carroll M. Harmon, P. Benson Ham

**Affiliations:** 1 Department of Surgery, Jacobs School of Medicine and Biomedical Sciences, University at Buffalo, Buffalo, NY 14215 USA; 2 Division of Pediatric Surgery, Jacobs School of Medicine and Biomedical Sciences, University at Buffalo, Buffalo, NY 14215 USA; 3 Jacobs School of Medicine and Biomedical Sciences, Buffalo, NY 14215 USA; 4 Division of Pediatric Surgery, John R. Oishei Children’s Hospital, Buffalo, NY 14203 USA; 5 MGH Institute of Health Professions, Boston, MA 02129 USA; 6 Department of Pediatric Surgery, Division of Pediatric Surgery, University at Buffalo, Jacobs School of Medicine and Biomedical Sciences, 1001 Main St, Buffalo, NY 14203 USA

**Keywords:** Esophageal atresia (EA), Tracheoesophageal fistula (TEF), Minimally invasive surgery (MIS), Thoracoscopic surgery, NSQIP, Pediatric surgery

## Abstract

**Purpose:**

Thoracoscopic repair of esophageal atresia (EA) and tracheoesophageal fistula (TEF) is a technically demanding operation. We hypothesize thoracoscopic repair of EA with TEF over time has increased, with improved or equivalent outcomes compared to open.

**Methods:**

NSQIP-P identified patients < 30 days with EA and TEF who underwent repair of both defects from 2016 to 2022. Patients were categorized into (1) intention to treat (thoracoscopic/thoracoscopic converted to open); and (2) thoracoscopic only, both compared to open only patients. Coarsened exact matching was performed. Pre/post-match analysis utilized *X*^2^, Fisher’s exact, and Mann-Whitney-U tests.

**Results:**

Overall, 1,188 patients were identified with 147 (12.4%) thoracoscopic only, 129 (10.8%) converted to open, and 912 (76.8%) open only. Thoracoscopic repair increased (2016–2022) for intention to treat (12.0% vs. 29.4%, *p* = 0.001) and thoracoscopic only (7.2% vs. 19.1%, *p* = 0.012) cohorts. Matched analysis identified thoracoscopic patients had reduced length of stay (*p* = 0.005) and longer operative time (< 0.01); however, no difference in other outcomes were identified (*p* > 0.05).

**Conclusion:**

Thoracoscopic repair of EA with TEF attempts have increased to roughly 30%, and it is associated with reduced length of stay and longer operative times without other outcome differences. This analysis affirms the short-term safety of thoracoscopic repair of EA and TEF.

**Level of evidence (I-V):**

Level III.

**Supplementary Information:**

The online version contains supplementary material available at 10.1007/s00383-026-06420-8.

## Introduction

Minimally invasive surgery (MIS) has been increasing in frequency in almost every field of surgery for the past several decades. Several benefits of MIS when compared to open procedures have been well-described including shorter recovery times, shorter hospital stays, less scarring, and better cosmetic results [1–5]; however, surgeons performing rare and more complex procedures have been slower to adopt an MIS approach, likely in part due to the lack of long-term research on their safety and efficacy as well as the rarity and technically demanding nature of certain conditions, limiting surgeon experience.

Repair of esophageal atresia (EA) with tracheoesophageal fistula (EA/TEF) has been slow to transition to a MIS approach. Though the first reported case of thoracoscopic approach for EA was in 1999 and EA/TEF in 2000, there continues to be a lack of consensus whether it is more beneficial compared to open repair [6, 7]. A 2016 meta-analysis comparing outcomes between open and thoracoscopic repair of EA/TEF reported that the thoracoscopic group had a longer operative time but shorter time to extubate, shorter time to first feed, and shorter length of hospital stay than the open repair group, and that there was no significant difference in complications such as leaks or strictures between the groups [1]. A 2021 single institution study reported no significant difference in operative time, presence of post-operative pneumothorax, anastomotic leakage, stricture development, or recurrent fistulas [8]. Additionally, a 2026 retrospective review of EA cases between 2020 and 2024 in Germany found no difference in length of hospital stay or cost between thoracoscopic or open [9]. There is some variability in the literature as another study reported an increased rate of reoperation in the thoracoscopic group compared to the open repair group [10]. Given the conflicting findings in prior studies, the aim of this analysis was to assess outcome differences between thoracoscopic and open repair of EA/TEF using more recent data. We hypothesized that the rate of thoracoscopic repair over time has increased, and with that increase, there are no outcome differences between the thoracoscopic and open approaches.

## Methodology

### Study population data source

The American College of Surgeons National Surgical Quality Improvement Program Pediatrics (ACS NSQIP-P) registry was utilized for this analysis [11]. NSQIP-P is a quality registry with over 150 participating hospitals contributing to the data collection with approximately 150 variables per surgical case [11, 12]. Patient outcomes are limited to 30-days; however, the data collection is robust, with trained NSQIP-P coders directly following up with patients and their families if follow-up data is unclear or missing.

### Study population

The ACS NSQIP-P registry was utilized to identify neonates between 1/1/2016 until 12/31/2022 with EA and TEF who underwent surgical repair at or before 30 days of age. Patients were identified using diagnosis codes (ICD-10) and procedural codes (CPT) to confirm a diagnosis of esophageal atresia with tracheoesophageal fistula (ICD-10: Q39.1). The associated thoracic repair of both defects was required for inclusion in analysis (CPT = 43312 or 43314: esophagoplasty, thoracic approach, with repair of tracheoesophageal fistula or esophagoplasty for congenital defect, thoracic approach, with repair of congenital tracheoesophageal fistula, respectively). Data retrieval started in 2016 as this was the first year diagnostic ICD-10 codes for EA/TEF were used. Any patient older than 30 days of age or who underwent EA or TEF repair alone was excluded. The age cutoff was chosen to exclude patients undergoing delayed repairs or revisions at other institutions, which could be counted separately within NSQIP-P, consistent with prior analyses [5].

Two sets of cohorts were created for analysis comparing the thoracoscopic and open approaches based on (1) intention to treat or (2) excluding conversion to open. Of note, the NSQIP-P database categorizes thoracoscopic interventions under the “Laparoscopic/MIS” category. The intention to treat thoracoscopic cohort was comprised of: “Laparoscopic/MIS” and “Laparoscopic/MIS Converted to Open” patients. The excluding conversion to open thoracoscopic cohort was comprised only of “Laparoscopic/MIS” cases. The open cohort used in both analyses were comprised of “Open, only” surgical patients. Within both analyses (intention to treat and excluding conversion to open), a new variable was created using the combination of reoperation and reported CPT codes to create a ‘Related Reoperation’ variable. Patients undergoing non-related surgical interventions were excluded from this new variable; however, the excluded operations from ‘Related Reoperation’ were included in the 30-day all reoperations variable. A full list of excluded reoperation CPT codes can be found in Appendix A. Four additional variables for both analyses were created: Diagnostic Scopes, Therapeutic Scopes, Serious Complications, and All Complications. *Diagnostic Scopes* were identified by reoperation associated with CPT codes relating to a diagnostic endoscopy, bronchoscopy or laryngoscopy. *Therapeutic Scopes* were identified by reoperation-associated CPT codes relating to an endoscopy, bronchoscopy or laryngoscopy with associated intervention (dilation, stent placement, etc.) (Appendix A). *Serious Complications* were defined as 30-day mortality, readmission, reoperation, bleeding events requiring transfusion, post-operative sepsis or septic shock, cardiac arrest, unplanned intubation, pneumonia, and organ space level surgical site infections (SSI). *All Complications* were defined as serious complications with the addition of deep incisional level SSI, superficial SSI, and wound dehiscence.

### Data analysis

#### Developing matched datasets

A coarsened exact matching (CEM) based on age, weight, cardiac risk factors (CRF), American Society of Anesthesiologists (ASA) classification, prematurity status (< 37 weeks gestational age at birth), race, and sex was completed for both the intention to treat and excluding conversion to open datasets using the MatchIt package in R [13–15].

#### MIS versus open analysis

Bivariate and multivariate analyses were performed on the unmatched and matched data sets for both the intention to treat and excluding conversion to open cohorts separately, including demographic, clinical characteristics, and outcomes using Mann-Whitney U for continuous variables, and χ2 and Fisher’s exact tests for categorical data when appropriate. Significance was defined as a *p* value with a two-sided significance level less than 0.05. Data analysis was performed in the statistical software R 4.4.2 (R Core Team, Vienna, Austria, 2024).

This project was reviewed and determined to be non-human subjects research by the Institutional Review Board of the principal investigator (STUDY00007840).

## Results

### Demographics, clinical characteristics, and MIS utilization over time

A total of 1,188 neonates with EA/TEF met inclusion criteria between 2016 and 2022, with 147 (12.4%) thoracoscopic only, 129 (10.8%) converted to open, and 912 (76.8%) open only patients.

#### Intention to treat

**Fig. 1:**
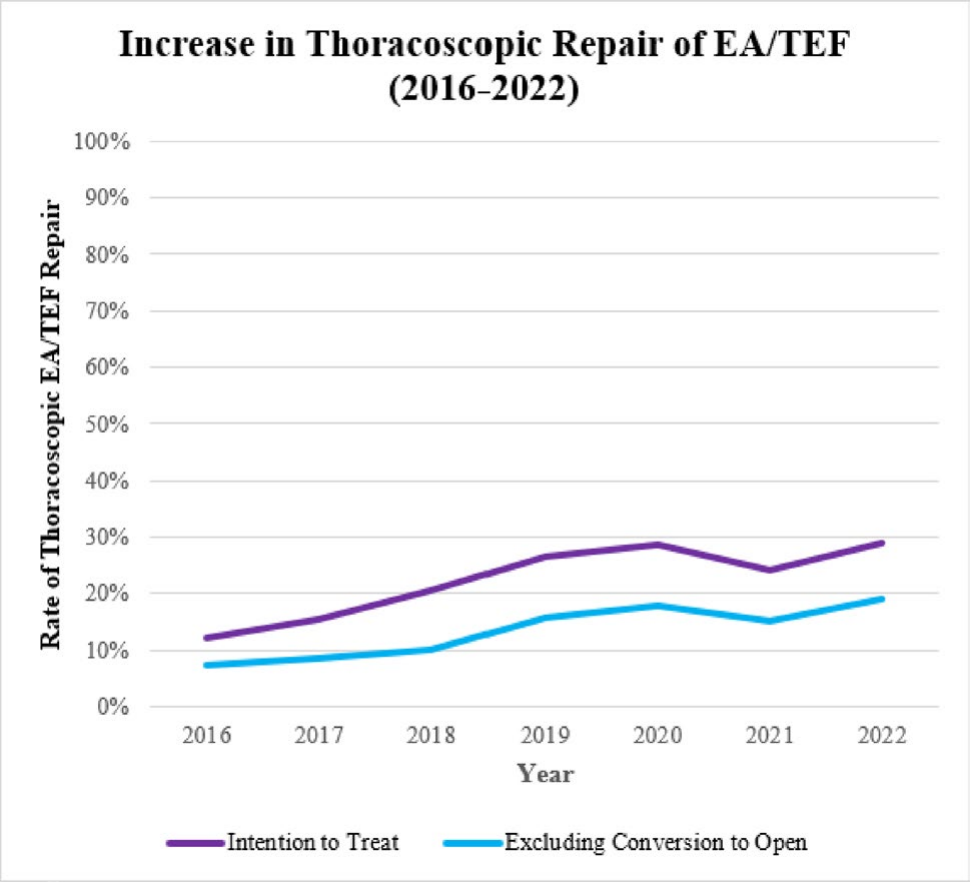
Rate of Thoracoscopic repair for Esophageal Atresia and Tracheoesophageal Fistula over Time. The absolute number of repairs per year in 2016 is 116 (9.8%), in 2017 is 154 (13.0%), in 2018 is 170 (14.3%), in 2019 is 159 (13.4%), in 2020 is 195 (16.4%), in 2021 is 191 (16.1%), and in 2022 is 203 (17.1%)

The rate of thoracoscopic EA/TEF repair in an intention to treat analysis compared to open has increased from 12.1% in 2016 to 29.1% in 2022 (*p* = 0.001) (Fig. 1). For demographic and clinical characteristic differences: patients who underwent MIS repair weighed more (2.64 kg vs. 2.54 kg, *p* = 0.021), were less often pre-term (27.5% vs. 38.1%, *p* = 0.002) and were less often ventilator dependent at the time of surgery (17.0% vs. 26.8%, *p* = 0.001). Otherwise, there were no other preoperative differences (Table 1).

**Table 1:** Pre-Operative Demographics and Clinical Characteristic Differences of Patients Undergoing Esophageal Atresia (EA) and Tracheoesophageal Fistula (TEF) Repair between Thoracoscopic and Open Approaches with and without Intention to Treat

Demographics & Clinical Characteristics	Open	Intention to Treat: Thoracoscopic	*p*-value	Excluding Conversion to Open: Thoracoscopic	*p*-value
N	912 (76.8%)	276 (23.2%)	-	147 (13.9%)	-
Age (days), mean (SD)*^+^	2.32 (2.10)	2.30 (2.36)	0.529	2.44 (2.45)	0.705
Weight (kg),mean (SD)* ^+^	2.54 (0.76)	2.64 (0.64)	**0.021**	2.71 (0.64)	**0.006**
Preterm**^+^	342 (38.1%)	76 (27.5%)	**0.002**	38 (25.9%)	**0.006**
Female, n (%)**^+^	398 (43.6%)	118 (42.8%)	0.848	66 (44.9%)	0.845
White, (%)***^+^	522 (57.2%)	162 (58.7%)	0.728	88 (59.9%)	0.190
Ventilator Dependance**	244 (26.8%)	47 (17.0%)	**0.001**	25 (17.0%)	**0.016**
Cardiac Risk Factors***^+^			0.109		0.166
None	136 (14.9%)	50 (18.1%)		29 (19.7%)	
Minor	208 (22.8%)	76 (27.5%)		39 (26.5%)	
Major	547 (60.0%)	143 (51.8%)		78 (53.1%)	
Severe	21 (2.3%)	7 (2.5%)		1 (0.7%)	
30-day Steroid Use***	9 (1.0%)	3 (1.1%)	~ 1.000 ^a^	0 (0.0%)	0.621
Hematologic disorder**	63 (6.9%)	14 (5.1%)	0.344	7 (4.8%)	0.428
ASA Classification**^+^			0.076		**0.026**
1–3	457 (50.4%)	156 (56.7%)		89 (60.5%)	
4–5	450 (49.6%)	119 (43.3%)		58 (39.5%)	

American Society of Anesthesiologists Classification System: ASA Class 1 – normal, healthy patient; ASA Class 2 – patient with mild systemic disease; ASA Class 3 – patient with severe systemic disease; ASA Class 4 – patient with severe systemic disease which is a constant threat to life. *Mann Whitney U Test. **Chi-square test. ***Fisher exact test. ^**+**^Variables used in coarsened exact matching (CEM). Bold text denotes statistical significance

#### Excluding conversion to open

The rate of successfully completed thoracoscopic EA/TEF repair compared to open patients has increased from 7.2% in 2016 to 19.1% in 2022 (*p* = 0.012) (Fig. 1). For demographic and clinical characteristic differences: patients who underwent MIS repair weighed more (2.71 kg vs. 2.54 kg, *p* = 0.006), were less often pre-term (25.9% vs. 38.1%, *p* = 0.006), ventilator dependent at the time of surgery (17.0% vs. 26.8%, *p* = 0.016), and ASA class 4–5 (39.5% vs. 49.6%, *p* = 0.026). Otherwise, there were no other preoperative differences (Table 1).

### Unmatched outcomes analysis

#### Intention to treat

In the unmatched outcome analysis, patients undergoing thoracoscopic intervention had longer operative times (227 min vs. 194 min, *p* < 0.001) and higher rates of overall reoperations (18.1% vs. 12.9%, *p* = 0.039), but no significant difference in related reoperations, and a shorter time from operation to discharging home (18 days vs. 20 days, *p* = 0.005). There was a significantly higher rate of diagnostic scopes post-operatively (4.7% vs. 2.0%, *p* = 0.022) in the thoracoscopic cohort compared to open. Otherwise, there were no significant unmatched outcome differences (Table 2).

**Table 2:** Unmatched Post-Operative Outcome differences for Patients undergoing Esophageal Atresia (EA) and Tracheoesophageal Fistula (TEF) Repair between Thoracoscopic and Open Approaches with and without Intention to Treat

Post-Operative Outcomes	Open	Intention to Treat: Thoracoscopic	*p*-value	Excluding Conversion to Open: Thoracoscopic	*p*-value
N	912 (76.8%)	276 (23.2%)	-	147 (13.9%)	-
Operative Time,min*	194.27 (77.60)	227.96 (82.64)	**< 0.001**	227.56 (84.20)	**< 0.001**
Days from Operation to Discharge*	20.31 (11.69)	18.13 (10.70)	**0.005**	16.99 (10.45)	**< 0.001**
Organ Space Infection**	34 (3.7%)	14 (5.1%)	0.413	9 (6.1%)	0.254
Unplanned Intubation**	124 (13.6%)	37 (13.4%)	~ 1.000^a^	18 (12.2%)	0.752
Transfusion**	110 (12.1%)	29 (10.5%)	0.551	11 (7.5%)	0.139
All Readmissions 30-day**	16 (1.8%)	9 (3.3%)	0.149	6 (4.1%)	0.127
All Reoperations 30-day**	118 (12.9%)	50 (18.1%)	**0.039**	31 (21.1%)	**0.011**
Reoperation Related 30-day**	97 (10.9%)	40 (15.0%)	0.084	28 (19.4%)	**0.005**
Diagnostic Scopes 30-day**	18 (2.0%)	13 (4.7%)	**0.022**	10 (6.8%)	**0.002**
Therapeutic Scopes 30-day**	23 (2.5%)	10 (3.6%)	0.443	9 (6.1%)	**0.035**
Serious Complications**	328 (36.0%)	104 (37.7%)	0.654	56 (38.1%)	0.685
All Complications**	359 (39.4%)	110 (39.9%)	0.940	60 (40.8%)	0.808
Mortality 30-day***	17 (1.9%)	5 (1.8%)	~ 1.000^a^	2 (1.4%)	~ 1.000^a^

*Mann Whitney U Test. **Chi-square test. ***Fisher exact test. ^a^ Indicates the p-value is approaching 1, and R rounded to report a value of 1. Of note this outcome analysis is performed on unmatched data with significant preoperative differences as seen in Table 1, and thus may be influenced by selection bias. Bold text denotes statistical significance

#### Excluding conversion to open

In the unmatched outcome analysis, patients successfully undergoing thoracoscopic intervention had longer operative times (228 min vs. 194 min, *p* < 0.001), shorter postoperative length of stay (16.99 vs. 20.31 days, *p* < 0.001), higher rates of overall reoperations (21.1% vs. 12.9%, *p* = 0.011), related reoperations (19.4% vs. 10.9%, *p* = 0.005), diagnostic scopes postoperatively (6.8% vs. 2.0%, *p* = 0.022), and therapeutic scopes postoperatively (6.1% vs. 2.5%, *p* = 0.035). Otherwise, there were no significant unmatched outcome differences (Table 2).

### Matched outcome analysis

#### Intention to treat

CEM on age, weight, cardiac risk factors (CRF), ASA classification, prematurity (< 37 wks), race, and sex yielded a matched dataset with 93 thoracoscopic patients and 193 open patients for analysis. When assessing for outcome differences between the thoracoscopic and open cohorts, thoracoscopic patients continued to have longer operative times compared to open (220 vs. 192, *p* = 0.009) consistent with the unmatched analysis; however, there were no significant differences in rates of reoperation, related reoperation, diagnostic scopes, or other outcome variables (Table 3).

**Table 3:** Coarsened Exact Matched Post-Operative Outcome differences for Patients undergoing Esophageal Atresia (EA) and Tracheoesophageal Fistula (TEF) Repair between Minimally Invasive Surgical (MIS) and Open Approaches with and without Intention to Treat

Post-Operative Outcomes	Open	Intention to Treat: MIS	*p*-value	Excluding Conversion to Open: MIS	*p*-value
N	193	93	–	50	-
Operative Time, min*	192.15 (72.81)	220.71 (88.23)	**0.009**	221.86 (84.71)	0.086
Days from Operation to Discharge*	18.33 (8.52)	17.84 (10.80)	0.135	15.70 (8.29)	**0.012**
Organ Space Infection***	4 (2.1%)	2 (2.2%)	~ 1.000^a^	0 (0.0%)	0.552
Unplanned Intubation***	14 (7.3%)	3 (3.2%)	0.285	1 (2.0%)	0.172
Transfusion***	11 (5.7%)	2 (2.2%)	0.234	0 (0.0%)	0.180
All Readmissions 30-day**	7 (3.6%)	6 (6.5%)	0.363	4 (8.0%)	0.209
All Reoperations 30-day***	15 (7.8%)	5 (5.4%)	0.622	4 (8.0%)	0.466
Reoperation Related 30-day***	15 (7.8%)	5 (5.4%)	0.622	4 (8.0%)	0.466
Diagnostic Scopes 30-day***	2 (1.0%)	4 (4.3%)	0.090	3 (6.0%)	0.094
Therapeutic Scopes 30-day***	4 (2.1%)	1 (1.1%)	~ 1.000^a^	1 (2.0%)	0.317
Serious Complications**	41 (21.2%)	15 (16.1%)	0.389	7 (14.0%)	0.737
All Complications**	45 (23.3%)	18 (19.4%)	0.545	9 (18.0%)	0.692
Mortality 30-day	0 (0.0%)	0 (0.0%)	-	0 (0.0%)	-

*Mann Whitney U Test. **Chi-square test. ***Fisher exact test. ^a^ Indicates the p-value is approaching 1, and R rounded to report a value of 1. Bold text denotes statistical significance

#### Excluding conversion to open

CEM on age, weight, cardiac risk factors (CRF), ASA classification, prematurity (< 37 wks), race, and sex yielded a matched dataset with 50 thoracoscopic patients and 108 open patients for analysis. When assessing outcome differences between the thoracoscopic and open cohorts, thoracoscopic patients had longer operative times compared to open, but this was not significant (222 vs. 190 min, *p* = 0.086). Thoracoscopic patients were discharged sooner than open patients, which was statistically significant (15.70 vs. 18.71 days, *p* = 0.012). There were no significant differences in rates of reoperation, related reoperation, diagnostic scopes, or other outcome variables (Table 3).

## Discussion

This analysis represents a comprehensive review of thoracoscopic compared to open esophageal and tracheoesophageal fistula repair. Using both an intention to treat and excluding conversion to open analysis with matching techniques, we compared findings of thoracoscopic and open cohorts. In our matched analyses, we found decreased length of stay (excluding conversion to open) and increased operative times (intention to treat) for thoracoscopic patients without other postoperative outcome differences.

The benefits and potential risks of thoracoscopic repair of this constellation of congenital defects remain an ongoing debate. Other analyses using large patient databases have been performed comparing thoracoscopic and open repair of EA/TEF patients, however they have used different methodology or older data. One analysis that found no difference in 30-day outcomes between the thoracoscopic and open cohorts had multiple preoperative differences between the two cohorts in their dataset, and did not use matching or other statistical methods to control for these differences prior to outcomes analysis [16]. A concern with pre-operative differences is the possibility that the reported outcomes were influenced by selection bias if larger, healthier patients proceeded to thoracoscopic versus smaller, sicker patients proceeding to open surgical repair. Thus, accounting for these preoperative differences is imperative. Another analysis comparing thoracoscopic and open cohorts with an intention to treat model utilized propensity score matching to control for preoperative differences and found higher rates of 30-day re-intervention for the thoracoscopic cohort [10]. Our analyses within both of our matched analyses (intention to treat and excluding conversion to open), using more recent data, contradicts this finding suggesting that the increased adoption and refinement of thoracoscopic techniques when applied to EA/TEF repair may have improved outcomes in recent years [10]. Within our unmatched analyses we did find a difference in reoperations, specifically a high rate of diagnostic scopes in both datasets and therapeutic scopes in the excluding conversion to open dataset. As stated earlier in the matched analysis, there were no significant postoperative outcome differences, including in all reoperations, diagnostic scopes, therapeutic scopes, and related reoperations for either the intention to treat or excluding conversion to open cohort. This suggests that when controlling for pre-operative factors, there are no short-term negative outcomes for the thoracoscopic cohort. We hypothesize that these findings in the unmatched analysis are due to the patients in the unmatched data including the larger, healthier thoracoscopic patients who discharged sooner (and were otherwise excluded in the matched analysis). These excluded patients would have had more time for scheduled postoperative interventions including endoscopic evaluation and dilation after early discharge from thoracoscopic repair to be captured within NSQIP-P’s 30-day postoperative complication variables, potentially skewing the unmatched results. Surgeons who do thoracoscopic repairs may be more likely to do earlier diagnostic or therapeutic scopes earlier than 30 days. This is interesting with regard to findings from the Midwest Pediatric Surgery Consortium, who evaluated EA/TEF repair at their participating institutions and followed dilations and stricture rates for a year postoperatively; they found no difference in dilations between the thoracoscopic and open cohorts, with the majority of strictures being diagnosed > 30 days postoperatively [17]. Our analysis in comparison to prior literature suggests that the increase in thoracoscopic utilization up to 29% for intention to treat and 19% for excluding conversion to open has likely driven down early complications for these patients, as the link between thoracoscopic experience and complication rates has been well established for EA/TEF [18].

There were some slight differences in the outcomes between the intention to treat and excluding conversion to open analysis. The main difference was the postoperative length of stay. In the intention to treat analysis, the noted shorter length of stay for the thoracoscopic patients compared to the open patients was not statistically significant. However, after exclusion of the converted to open patients, a significant difference was found. This could be explained by the almost 50% exclusion as just under half of the intention to treat thoracoscopic cohort were converted to open, which would prolong length of stay for many of the patients. This association of longer length of stay for thoracotomy compared to thoracoscopy has been seen in other pediatric thoracoscopic surgeries including congenital lung malformations [19], and more broadly across multiple pediatric surgeries [20].

A consistent finding across these analyses, which was also identified in ours, is the longer operative time for the minimally invasive approach [10, 16]. It is possible that the technical complexity of suturing in a small space with the esophagus on tension is difficult and can potentially lead to increased OR times, anesthesia exposure and cost to the patient. Interestingly, the exclusion of patients converted to open did not decrease operative time in this analysis, which we hypothesize is due to conversions likely occurring after the upper esophagus dissection and before or during the sutured anastomosis of the esophagus. The increased time it takes many surgeons to suture thoracoscopically could result in a similar overall operative time between completing the anastomosis thoracoscopically vs. converting to open to start or complete the anastomosis via a thoracotomy. Increased operative time is a common finding in comparisons between MIS and open approaches and should be put into the broader context of MIS and open surgery. Specifically for EA/TEF repair, there are multiple additional benefits reported in the literature to proceeding with thoracoscopic repair including shorter time to extubate, shorter time to first feed, and shorter length of hospital stay than the open repair group [1, 2]. In addition, outside of EA/TEF repair, there is the long-term benefit of avoiding a thoracotomy incision in general, which is often associated with scoliosis and rib deformities [3–5]. The additional benefits of a minimally invasive approach, in combination with our study findings, lead us to recommend the MIS approach for surgeons who have the skillset and are comfortable performing it.

## Limitations

There are multiple limitations to our analysis. We did not address long-term complications as NSQIP-P data is limited to 30-days post-operation. One of the major concerns for the MIS approach is the potentially higher anastomotic stricture rate as identified by prior individual studies [2]; however, other individual institutional studies [8], an analysis of the patients in the Midwest Pediatric Surgery Consortium [17], and meta-analyses have aggregated additional data and not found this association for thoracoscopic vs. Open EA/TEF repair patients [1, 21]. Additionally, data used in this analysis is retrospective and can lead to limitations in the granularity of the data available for analysis. Specifically, we did not have data related to gap length (and if a patient had long-gap EA), the tension on the anastomosis, or the surgeon’s and/or institution’s experience with thoracoscopic repair and thus we were unable to specifically control for these variables within our analysis. In addition, NSQIP-P does not provide additional context related to why a surgery was converted to open. In relation to operative time, information related to case complexity, trainee involvement, or operative setup is not available, which limits our conclusions. Lastly, while propensity score matching is a valid statistical tool for generating matched data cohorts, due to the concerns of thoracoscopic patients being generally healthier than their thoracotomy counterparts for EA/TEF, we elected to perform coarsened exact matching, which assesses both cohorts of patients for exact matches, to have more ‘exact’ cohorts for our matched analysis [13].

## Conclusion

Thoracoscopic repair of esophageal atresia with tracheoesophageal fistula attempts have increased to roughly 30% of repairs and is associated with longer operative times and reduced length of stay without other outcome differences. Our analysis affirms the short-term safety of thoracoscopic repair of EA and TEF as it has increased in frequency.

## Supplementary Information

Below is the link to the electronic supplementary material.

Supplementary Material 1

## Data Availability

The data utilized in this analysis is from the National Surgical Quality Improvement Program Pediatric registry. It can be accessed after obtaining permission from the American College of Surgeons with a Participant Use Data File (PUF) request.

## Abbreviations

ACS NSQIP-P: American College of Surgeons National Surgical Quality Improvement Program Pediatric
ASA: American society of anesthesiologists classification
CEM: Coarsened exact matching
CPT: Current procedural terminology
CRF: Cardiac risk factors
EA: Esophageal atresia
ICD 10: International classification of diseases, tenth revision, clinical modification
LOS: Length of stay
SSI: Surgical site infection
MIS: Minimally invasive surgery
TEF: Tracheoesophageal fistula

## References

[1] Yang YF, Dong R, Zheng C, Jin Z, Chen G, Huang YL et al (2016) Outcomes of thoracoscopy versus thoracotomy for esophageal atresia with tracheoesophageal fistula repair: a PRISMA-compliant systematic review and meta-analysis. Medicine Baltimore 95(30):e4428. 10.1097/md.0000000000004428 27472740 10.1097/MD.0000000000004428 PMC5265877

[2] Yalcin S, Bhatia AM, He Z, Wulkan ML (2024) Short- and long-term outcomes of thoracoscopic and open repair for esophageal atresia and tracheoesophageal fistula. J Pediatr Surg 59(12):1616. 10.1016/j.jpedsurg.2024.08.002 10.1016/j.jpedsurg.2024.08.002 39218728

[3] Aubert O, Lacher M, Mayer S, Frahm J, Voit D, Rosolowski M et al (2024) Increased musculoskeletal deformities and decreased lung volume in patients after Ea/Tef repair - a real-time Mri study. Ann Surg. 10.1097/sla.0000000000006193 38328992 10.1097/SLA.0000000000006193

[4] Hattori K, Kawashima H, Ishimaru T, Yanagida Y, Miyake K, Iguchi M et al (2024) Musculoskeletal deformities after thoracoscopic versus conventional open repair for esophageal atresia. Asian J Surg 47(2):968–72. 10.1016/j.asjsur.2023.11.043 38030485 10.1016/j.asjsur.2023.11.043

[5] Borselle D, Grochowski K, Gerus S, Międzybrodzki K, Kołtowski K, Jasińska A et al (2024) Thoracic musculoskeletal deformities following surgical treatment of esophageal atresia - thoracoscopic versus open approach: a retrospective two centers cohort study. J Pediatr Surg 59(9):1719–24. 10.1016/j.jpedsurg.2024.03.023 38594136 10.1016/j.jpedsurg.2024.03.023

[6] Lobe TE, Rothenberg S, Waldschmidt J, Stroedter L (1999) Thoracoscopic repair of esophageal atresia in an infant: a surgical first. Pediatr Endosurgery Innovative Techniques 3(3):141–148. 10.1089/pei.1999.3.141

[7] Rothengerg SS (2000) Thoracoscopic repair of a tracheoesophageal fistula in a newborn infant. Pediatr Endosurgery Innovative Techniques 4(4):289–294. 10.1089/pei.2000.4.289

[8] Yang S, Wang P, Yang Z, Li S, Liao J, Hua K et al (2021) Clinical comparison between thoracoscopic and thoracotomy repair of Gross type C esophageal atresia. BMC Surg 21(1):403. 10.1186/s12893-021-01360-7 34809633 10.1186/s12893-021-01360-7 PMC8607600

[9] Wennemann L, Blaser J, Wiesner S, Zeidler J, Obed M, Weidner J et al (2026) Esophageal Atresia Repair in Germany: Utilization Patterns, Hospital Characteristics and Costs. Eur J Pediatr Surg 36(1):29–35. 10.1055/a-2676-2933 40840508 10.1055/a-2676-2933

[10] Castro P, Fall F, Pace D, Mack SJ, Rothstein DH, Devin CL et al (2024) Association of Operative Approach With Postoperative Outcomes in Neonates Undergoing Surgical Repair of Esophageal Atresia and Tracheoesophageal Fistula. J Pediatr Surg 59(11):161641. 10.1016/j.jpedsurg.2024.07.026 39147683 10.1016/j.jpedsurg.2024.07.026

[11] ACS NSQIP Pediatric Participant Use Data File (2024) https://www.facs.org/quality-programs/data-and-registries/pediatric/participant-use-data-file/; 2024

[12] User Guide for the 2021 ACS NSQIP Pediatric Surgical Antibioitc Prophylaxis Participant Use Data File (SAP PUF) (2023) American College of Surgeons Website. Amercian College of Surgeons, p 12

[13] King G, Nielsen R (2019) Why Propensity Scores Should Not Be Used for Matching. Political Anal 27(4):435–454. 10.1017/pan.2019.11

[14] Harfouche MN, Feliciano DV, Kozar RA, DuBose JJ, Scalea TM (2023) A Cautionary Tale: The Use of Propensity Matching to Evaluate Hemorrhage-Related Trauma Mortality in the American College of Surgeons TQIP Database. J Am Coll Surg 236(6):1208–1216. 10.1097/xcs.0000000000000669 36847370 10.1097/XCS.0000000000000669

[15] Ho D, Imai K, King G, Stuart EA (2011) MatchIt: Nonparametric Preprocessing for Parametric Causal Inference. J Stat Softw 42(8):1–28. 10.18637/jss.v042.i08

[16] Etchill EW, Giuliano KA, Boss EF, Rhee DS, Kunisaki SM (2021) Association of operative approach with outcomes in neonates with esophageal atresia and tracheoesophageal fistula. J Pediatr Surg 56(12):2172–2179. 10.1016/j.jpedsurg.2021.04.006 33994203 10.1016/j.jpedsurg.2021.04.006

[17] Marquart JP, Bowder AN, Bence CM, St Peter SD, Gadepalli SK, Sato TT et al (2023) Thoracoscopy versus thoracotomy for esophageal atresia and tracheoesophageal fistula: Outcomes from the Midwest Pediatric Surgery Consortium. J Pediatr Surg 58(1):27–33. 10.1016/j.jpedsurg.2022.09.015 36283849 10.1016/j.jpedsurg.2022.09.015

[18] Kim W, Son J, Lee S, Seo JM (2020) The learning curve for thoracoscopic repair of esophageal atresia with distal tracheoesophageal fistula: A cumulative sum analysis. J Pediatr Surg 55(11):2527–2530. 10.1016/j.jpedsurg.2020.06.005 32646663 10.1016/j.jpedsurg.2020.06.005

[19] Adams S, Jobson M, Sangnawakij P, Heetun A, Thaventhiran A, Johal N et al (2017) Does thoracoscopy have advantages over open surgery for asymptomatic congenital lung malformations? An analysis of 1626 resections. J Pediatr Surg 52(2):247–51. 10.1016/j.jpedsurg.2016.11.014 27889066 10.1016/j.jpedsurg.2016.11.014

[20] Kiblawi R, Zoeller C, Zanini A, Kuebler JF, Dingemann C, Ure B et al (2022) Laparoscopic versus Open Pediatric Surgery: Three Decades of Comparative Studies. Eur J Pediatr Surg 32(1):9–25. 10.1055/s-0041-1739418 34933374 10.1055/s-0041-1739418

[21] Way C, Wayne C, Grandpierre V, Harrison BJ, Travis N, Nasr A (2019) Thoracoscopy vs. thoracotomy for the repair of esophageal atresia and tracheoesophageal fistula: a systematic review and meta-analysis. Pediatr Surg Int 35(11):1167–1184. 10.1007/s00383-019-04527-9 31359222 10.1007/s00383-019-04527-9

