# Supplementary material for: Trends and outcomes of thoracoscopic esophageal atresia and tracheoesophageal fistula repair: a retrospective analysis 2016–2022
Source: Pediatr Surg Int. 2026 Apr 15;42(1):195. doi: 10.1007/s00383-026-06420-8 (PMC13083507; doi:10.1007/s00383-026-06420-8)
Supplement: Supplementary file 1 — Supplementary Material 1 [file 383_2026_6420_MOESM1_ESM.docx]

Appendix A – Included and Excluded CPT Codes Associated with Author Generated Variables

*Diagnostic Scopes Included CPT Codes:*

43235 - Diagnostic esophagogastroduodenoscopy, flexible, transoral; including collection of specimen(s) by brushing or washing, when performed (separate procedure)

43200 - Diagnostic esophagoscopy, flexible; transoral; including collection of specimen(s) by brushing or washing, when performed (separate procedure)

43197 - Diagnostic esophagoscopy, flexible, trans nasal; including collection of specimen(s) by brushing or washing, when performed (separate procedure)

43191 - Diagnostic esophagoscopy, rigid, transoral; including collection of specimen(s) by brushing or washing when performed (separate procedure)

31627 - Diagnostic bronchoscopy with cell washing with computer assistance

31625 - Diagnostic bronchoscopy with cell washing with biopsy

31624 - Diagnostic bronchoscopy with cell washing with bronchoalveolar lavage

31623 - Diagnostic bronchoscopy with cell washing with brushings

31622 - Diagnostic bronchoscopy with cell washing

31615 - Diagnostic bronchoscopy through tracheostomy

31579 – Laryngoscopy with stroboscopy

31575 – Diagnostic laryngoscopy, flexible

31535 – Direct laryngoscopy with biopsy

31526 – Diagnostic direct laryngoscopy with or without tracheoscopy

31525 – Direct laryngoscopy for diagnostic procedure

31520 – Direct laryngoscopy on a newborn

31510 – Indirect laryngoscopy with biopsy

*Therapeutic Scopes Included CPT Codes:*

43453 - Dilation of esophagus; by unguided sound or bougie, single or multiple passes over guide wire

43450 – Dilation of esophagus; by unguided sound or bougie, single or multiple passes

43266 – Esophagogastroduodenoscopy with placement of endoscopic stent (includes pre and post dilation and guide wire passage, when performed)

43249 - Esophagogastroduodenoscopy with transendoscopic ballon dilation of esophagus (less than 30mm in diameter)

43233 – Flexible esophagogastroduodenoscopy with dilation of esophagus with ballon (30mm diameter or larger) includes fluoroscopic guidance, when performed

43226 - Flexible esophagoscopy with insertion of guidewire followed by passage of dilators over guide wire

43220 - Flexible esophagoscopy with transendoscopic ballon dilation (less than 30 mm diameter)

43214 - Flexible esophagoscopy with dilation of esophagus with balloon

43213 - Flexible esophagoscopy with dilation of esophagus by ballon or dilator, retrograde (includes fluoroscopic guidance, when performed)

43212 - Flexible esophagoscopy with placement of endoscopic stent (includes pre and post dilation and guidewire passage when performed)

43196 - Rigid esophagogastroduodenoscopy with ballon dilation (less than 30 mm diameter) with insertion of guidewire followed by dilation over guidewire

43195 - Rigid esophagogastroduodenoscopy with ballon dilation (less than 30 mm diameter)

31640 – Bronchoscopy with excision of tumor

31630 – Bronchoscopy with dilation

31541 – Direct laryngoscopy with excision of tumor and or stripping of vocal chords with microscope

31528 – Direct laryngoscopy with initial dilation

*Related Reoperations Excluded CPT Codes:*

30115 – Polypectomy nasal removal

30540 – Repair procedure on nose

30999 – Unlisted nasal surgery

31500 – Emergent intubation

33208 – Pacemaker placement

33506 – Repair of anomalous coronary artery from pulmonary artery origin

33545 – Repair of postinfarction ventricular septal defect with or without myocardial resection

33730 – Complete repair of anomalous venous return

40799 – Unlisted procedure, lips
